# Scrofula

**Published:** 1889-11-02

**Authors:** 


### SURGERY.

### SCROFULA.

"On the Treatment of Scrofulous Glands"* is the title of a recent paper by Mr. F. Treves, surgeon to the London Hospital. The author remarks that it has been shown at one period that scrofula and tuberculosis are identical conditions, while at another period it has been shown that they are fundamentally unlike. Some have believed that while tuberculosis is a disease of the general system, strumous manifestations are essentially local, while others have opined that both are general. The author rightly says all scrofulous manifestations are due to the tubercular process. It may be said that the phthisical lung, the white swelling, the carious spine, the tubercular testicle, and the scrofulous gland in the neck are all due to the same process, modified only by locality. The general treatment of a scrofulous gland should be as follows: The best hygienic conditions are required, and as regards a beneficial climate, Margate is mentioned. Next, clothing plays an important part, and the patient should be covered with wool. Iodine, in the form of Kreuznach waters, appears in many instances to be distinctly beneficial, but the author does not place any reliance on syrup of iodide of iron, or on sulphide of calcium, medicines which are so generally prescribed. As regards local treatment, *rest* is a primary factor, and for this purpose, when there are large glands in the neck, Mr. Treves recommends a peculiar homemade stock, or a "wood-wool" collar. Iodine paint is not recommended, but ointments often appear to have some good effect, more, however, attributable to rubbing than to the ointment itself. On the whole, the author appears to think simple water dressing is as good as any other application. When scrofulous glands resist such treatment and operative measures are necessary, excision is the simplest, safest, and most certain method. Mr. Treves has removed from one patient as many as "eighty-four glands, all of which were caseous, while not a few were suppurating." The glands should be cut out, not torn out. The wounds should, of course, follow as far as possible the natural lines on folds of the skin. All bleeding must be well arrested and the wound washed with an antiseptic solution. The edges must be carefully adjusted by sutures of silkworm gut. The part should be dressed with sponge only, and should be subjected to sufficient pressure to obliterate the cavity of the wound. The neck must be kept rigid for about ten days. The author remarks: "For no measures which have been employed for the treatment of the strumous neck, can such excellent results be claimed as attend on the simple excision of the glands. Considering the grave complications attending the disease, the tedious path it follows, and the disfigurement it leaves, it may be permitted to regard this operation as not the least improvement in modern surgery." Crushing, injection, the cautery, puncture, scraping out the softened substance from the gland, are operations which have been endorsed and practised for this disease. Others have recommended the expectant treatment of fomentations, poultices, ointments, liniments, or the like. Most authors draw a distinction between acute and *chronic lymphadenitis*, and a standard authority, with reference to the plan of removing enlarged glands, observes, "sometimes a gland which is steadily enlarging and softening in spite of treatment may be removed entire before the skin is involved, and rapid union obtained with little scarring." But it has remained for Mr. Treves to show that removal is the best treatment. We are not sanguine, however, that removal will become the general plan. Many practitioners, and more of the public, are adverse for various reasons to the use of the knife, and do not see sufficient danger in enlarged glands to justify operative procedure. There is little doubt, however, that what Mr. Treves says is perfectly correct and that the operation is an improvement in modern surgery.

* *Lancet*, Sept. 5, 1889.

